# Effect of theobromine on dissolution of uric acid kidney stones

**DOI:** 10.1007/s00345-022-04059-3

**Published:** 2022-06-11

**Authors:** Francesca Julià, Antonia Costa-Bauza, Francisco Berga, Felix Grases

**Affiliations:** Laboratory of Renal Lithiasis Research, University Institute of Health Sciences Research (IUNICS-IdISBa), University of Balearic Islands, 07122 Palma de Mallorca, Spain

**Keywords:** Theobromine, *N*-acetylcysteine, Uric acid renal calculi, Dissolution

## Abstract

**Purpose:**

Uric acid renal lithiasis has a high prevalence and a high rate of recurrence. Removal of uric acid stones can be achieved by several surgical techniques (extracorporeal shock wave lithotripsy, endoscopy, laparoscopy, open surgery). These stones can also be eliminated by dissolution within the kidneys, because the solubility of uric acid is much greater when the pH is above 6. At present, *N*-acetylcysteine with a urinary basifying agent is the only treatment proposed to increase the dissolution of uric acid stones. In this paper, we compare the effect of theobromine and *N*-acetylcysteine on the in vitro dissolution of uric acid calculi in artificial urine at pH 6.5.

**Methods:**

The dissolution of uric acid renal calculi was performed in a temperature-controlled (37 °C) chamber. A peristaltic pump was used to pass 750 mL of synthetic urine (pH 6.5) through a capsule every 24 h. Stone dissolution was evaluated by measuring the change in weight before and after each experiment.

**Results:**

*N*-acetylcysteine increased the dissolution of uric acid calculi, but the effect was not statistically significant. Theobromine significantly increased the dissolution of uric acid calculi. Both substances together had the same effect as theobromine alone. The addition of theobromine to a basifying therapy that uses citrate and/or bicarbonate is a potential new strategy for the oral chemolysis of uric acid stones.

**Conclusion:**

Theobromine may prevent the formation of new stones and increase the dissolution of existing stones.

## Introduction

Uric acid renal lithiasis has a high prevalence, in that it accounts for more than 10% of kidney stones, and a high rate of recurrence, in that an individual may form multiple stones within a single year [1, 2]. The increasing prevalence of obesity and metabolic syndrome may be responsible for the significant increase in uric acid renal lithiasis during recent years [3, 4]. Therefore, uric acid renal lithiasis is a common, significant, and serious public health problem. Uric acid renal lithiasis may be prevented by oral administration of urinary alkalinizers, such as citrate, and/or inhibitors of uric acid crystallization, such as theobromine (TB) [5]. Removal of existing uric acid stones can be achieved by several surgical techniques, including extracorporeal shock wave lithotripsy, endoscopy, laparoscopy, and open surgery. However, uric acid stones can also be eliminated noninvasively by dissolution within the kidneys, because the solubility of uric acid increases greatly at pH values above 6. Obviously, the dissolution rate depends on the size of the stone and its location in the kidney, and greater irrigation increases the rate of dissolution. Alkalinization of the urine can be achieved with high doses of citrate, which is sometimes accompanied by use of bicarbonate. However, in some cases the oral consumption of high doses of citrate or citrate and bicarbonate can lead to stomach discomfort, and can induce the formation of sodium urate shells on the uric acid stones if used for long periods [6]. Nevertheless, stone dissolution without surgery has clear advantages, despite not being widely used in clinical practice [7–10].

*N*-acetylcysteine (NAC) with a urinary basifying agent has been proposed to increase the dissolution of uric acid stones [11]. NAC is a mucolytic agent that acts by reducing the viscosity of bronchial secretions. It works by cleaving the disulfide bridges of mucoproteins, making them less viscous [12], although this combination is not recommended in the current guidelines for uric acid stones.

In this paper we compared the effect of TB and NAC on the in vitro dissolution of uric acid stones in synthetic urine.

## Materials and methods

### Reagents and solutions

Uric acid, TB, NAC and escin were from Sigma-Aldrich (St. Louis, MO, USA) and synthetic urine components were from Panreac (Montcada i Reixac, Barcelona, Spain). Chemicals of analytical reagent-grade purity were dissolved in ultra-pure deionized water from a Milli-Q system. A uric acid stock solution was prepared daily by dissolving 0.4 g/L uric acid with 1 M NaOH (final pH: 10.52). A solution of “concentrated” synthetic urine was prepared by dissolving double the amounts of all substances listed in Table 1. Calcium and oxalate were not included to prevent crystallization of calcium oxalate. The pH of this “concentrated” synthetic urine was adjusted to 6.20. During experiments, equal volumes of a uric acid solution and the “concentrated” synthetic urine were mixed, so the final concentration of uric acid was 0.2 g/L and the concentrations of other compounds were as indicated in Table 1.

**Table 1:** Composition of synthetic urine

Substance	Concentration (g/L)
Na_2_SO_4_·10H_2_O	3.12
MgSO_4_·7H_2_O	0.73
NH_4_Cl	2.32
KCl	6.07
NaH_2_PO_4_·2H_2_O	1.21
Na_2_HPO_4_·12H_2_O	2.80
NaCl	6.53
Uric acid	0.20

Final pH = 6.5

### Experimental procedure

Post-extracorporeal shock wave lithotripsy (ESWL) fragments of uric acid stones were selected from a collection of anonymous kidney stone samples from the Laboratory of Renal Lithiasis Research belonging to University Institute of Health Sciences Research of the University of Balearic Islands. This collection has been generated from the routine kidney stone diagnostic study service that the Laboratory of Renal Lithiasis Research performs for the hospitals of the Balearic Islands Community. Kidney stones were studied and classified using the general protocol adopted by our laboratory. This methodology includes the use of optical stereomicroscopy, infrared spectrometry and scanning electron microscopy (SEM) [13]. All selected fragments had similar morphology and size.

**Fig. 1:**
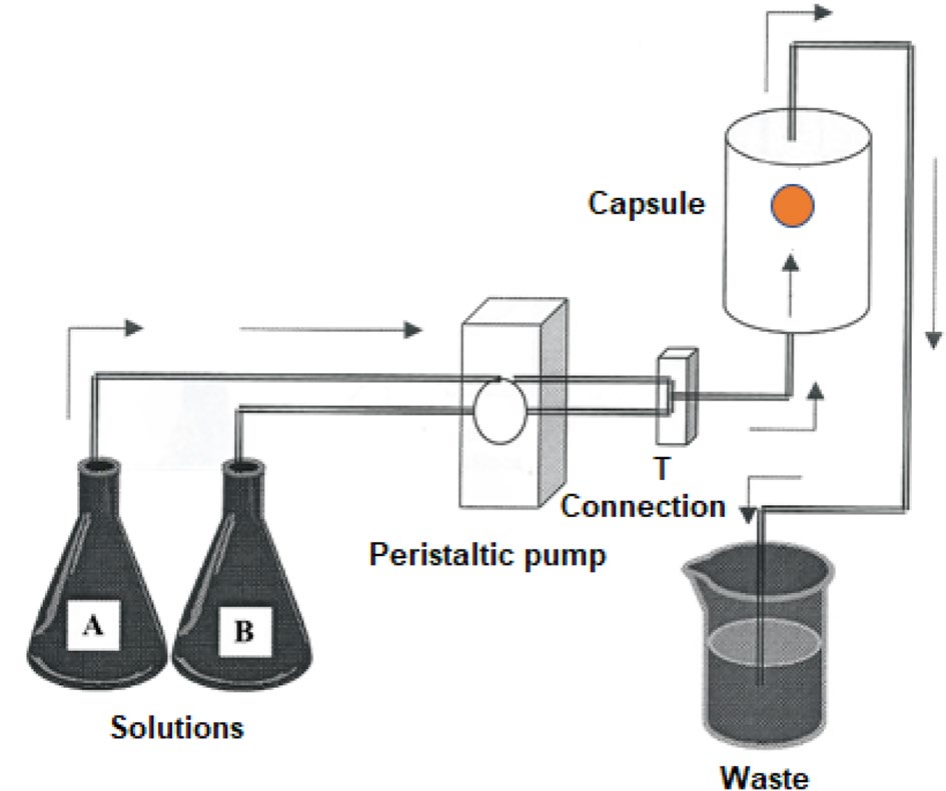
Experimental model used to examine the effect of different treatments on the dissolution of uric acid stones. See “Material and methods” for a description

In vitro dissolution of four uric acid calculi was performed simultaneously in a temperature-controlled chamber which remained at 37 °C during the course of experiments (48 h). In each experiment, four hermetic flow capsules (Fig. 1) were used, each containing 1 fragment of a uric acid calculus with no pre-treatment. A multichannel peristaltic pump was used to transfer the solution of “concentrated” synthetic urine (solution A; pH 6.20), with or without the experimental additive (see below) at a rate of 375 mL/day and a solution of 0.4 g/L of uric acid (solution B; pH 10.52) at the same rate. Both solutions were maintained at 37 °C and were mixed in a T connection before introduction into the capsule. Thus, 750 mL of synthetic urine (final pH: 6.5) passed through the capsule every 24 h. This is approximately the volume of urine that typically passes through a single human kidney each day.

The effects of adding 40 mg/L TB, 20 mg/L NAC, 40 mg/L escin or a mixture of 20 mg/L NAC with 40 mg/L TB (NAC + TB) on the dissolution of uric acid calculi were compared with the results obtained from controls treated with synthetic urine with no admixtures.

Experiments using a higher concentration of TB (80 mg/L) and an incubation period of 168 h with 40 mg/L of TB were also performed.

### Evaluation

The calculi were dried at 37 °C for 24 h before and after each experiment until they reached constant weight, determined using a precision balance. Fragment dissolution was calculated as the change in weight. Mean dissolution was determined and standardized by calculating the relative mass decrease, and thus did not consider the effect of surface area.

The morphological and structural characteristics of the samples, before and after dissolution, were observed using scanning electron microscopy (Hitachi S-3400 N) coupled with RX energy dispersive microanalysis (Bruker AXS XFlash Detector 4010).

### Statistics

The normality of data distributions was determined by inspection of plots. Data were presented as means with 95% confidence intervals (CIs). For continuous variables, 3 or more groups were compared using ANOVA with the Bonferroni post hoc correction, and 2 groups were compared using Student’s *t* test. A two-tailed *p* value less than 0.05 was considered statistically significant. Statistical analyses were performed using SPSS version 25.0 (SPSS Inc., Chicago, IL, USA).

## Results

**Fig. 2:**
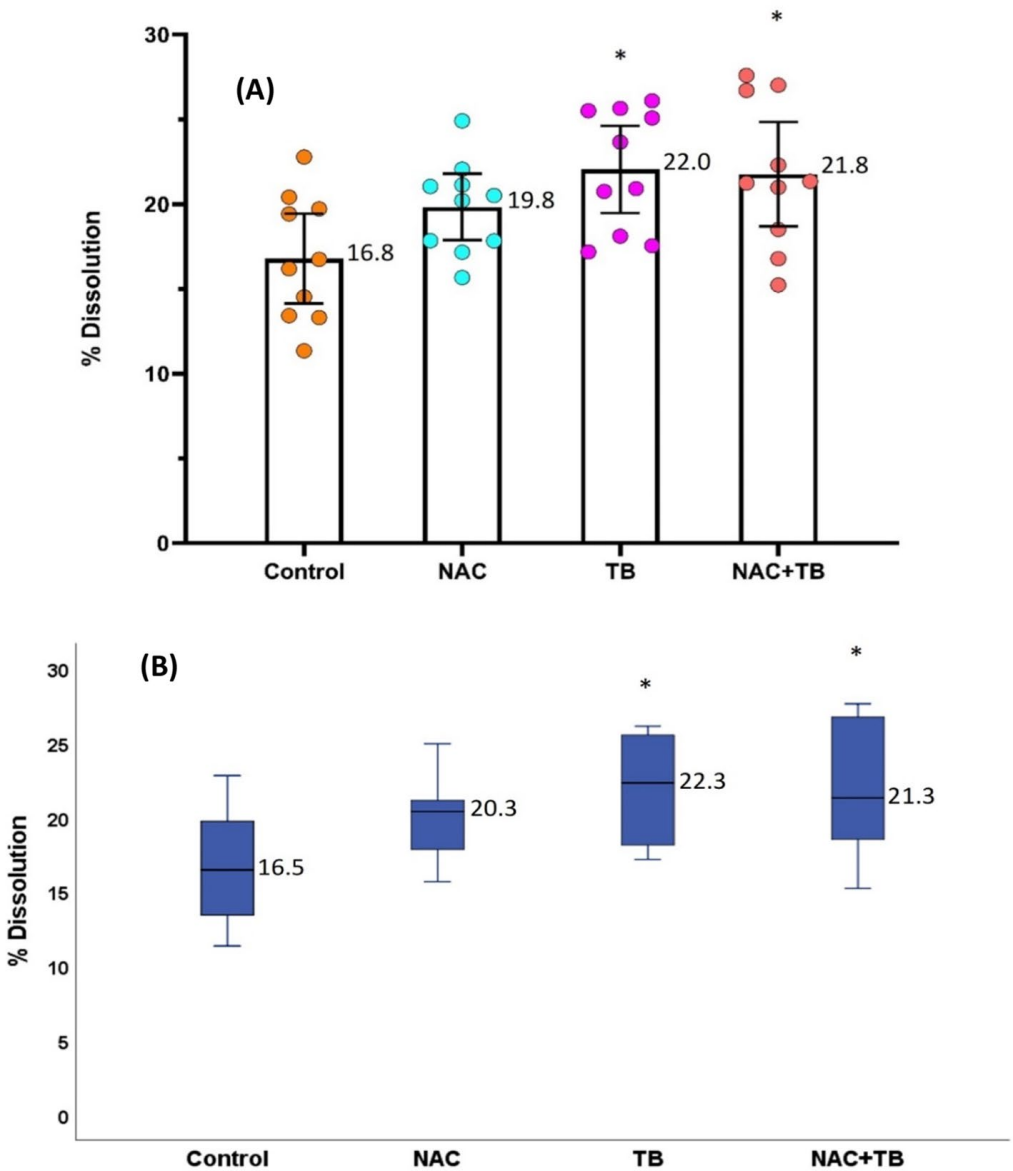
Effect of TB (40 mg/L), NAC (20 mg/L), and a mixture of NAC (20 mg/mL) + TB (40 mg/mL) on the dissolution of uric acid stones at pH 6.5. Percentage of dissolution was expressed as mean ± 95% CI **A** and as median ± interquartile range **B**, with 10 replicates per group. *Significantly different from the Control

**Fig. 3:**
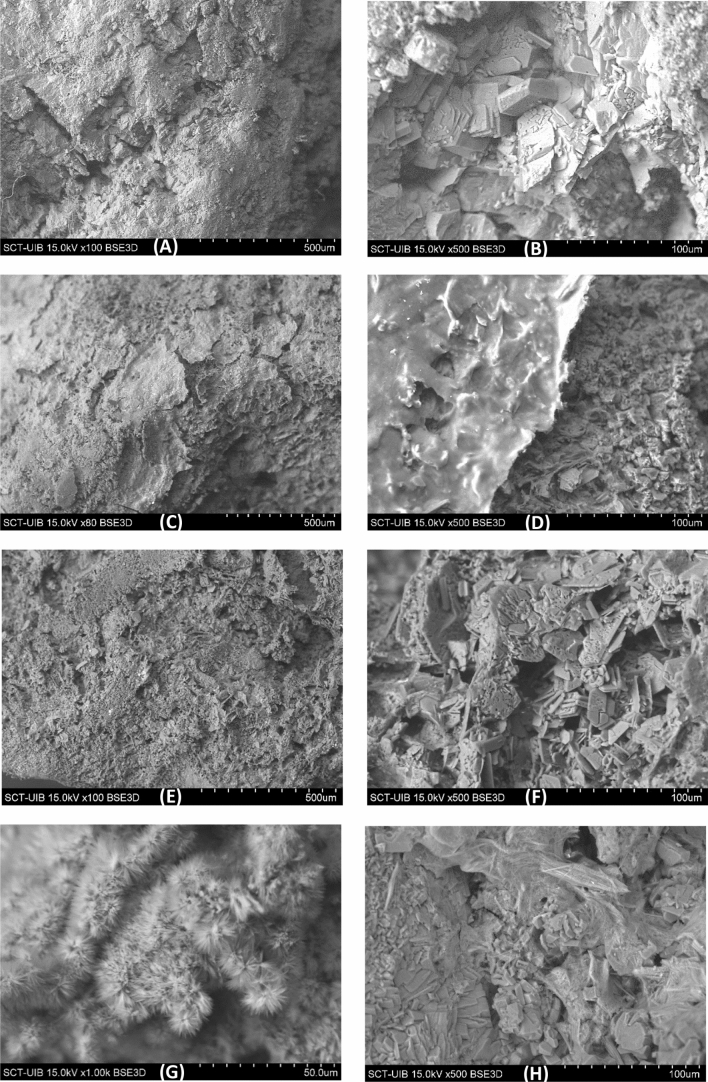
SEM images of uric acid stones. **A**, **B** Before dissolution: surface and higher magnification, showing compact uric acid crystals. **C**, **D** After NAC treatment: surface, showing detachment of the external layer of organic matter and higher magnification. **E**, **F** After TB treatment: surface and higher magnification, showing partial dissolution of uric acid crystals. **G** Sodium and potassium urate crystals on the surface of a uric acid stone after incubation in control solution for 168 h. **H** Sodium and potassium urate needle-like crystals formed “in vivo” on the surface of a uric acid stone

We first examined the effect of NAC, TB, and NAC + TB on the dissolution of uric acid stones in artificial urine (Fig. 2). Relative to the control, NAC treatment increased dissolution, although this effect was not statistically significant. TB treatment significantly increased dissolution, and the effect was similar for TB and NAC + TB. Thus, while treatment with NAC was not statistically significant, treatments with TB and NAC + TB were. Escin did not increase stone dissolution (data not shown). Notably, there was great variability among identically treated samples in these experiments (Fig. 2), presumably because of the different structures of the calculi. Obviously, the most porous stones dissolved at a higher rate than the most compact stones, but other factors can also affect dissolution, such as organic matter coating the stones (Fig. 3 A,B). Our SEM images showed that NAC facilitated the elimination of organic matter layers on the stones (Fig. 3 C,D) and that TB accelerated the dissolution of the uric acid crystals (Fig. 3 E,F). Despite their different effects, we observed no additive or synergistic effects when NAC and TB were used together.

When stones were incubated for 168 h, we identified areas with significant amounts of sodium and potassium urates (Fig. 3 G), such as those observed on the surface of some uric acid kidney stones (Fig. 3 H).

When we used a higher TB concentration (80 mg/L), the dissolution was greater than in the control (21.9 vs 17.1%), but was not significantly different from that obtained with the lower TB concentration.

## Discussion

TB is an alkaloid molecule in the xanthine family that occurs in the cocoa ‘bean’, and dark chocolate consists of about 1 to 4% TB [14]. TB is related to caffeine and theophylline, but it has weaker effects on the central nervous system than caffeine [15]. Due to the structural characteristics of TB, it can inhibit uric acid crystallization, especially when the urinary concentration is greater than 15 mg/L [16]. Thus, TB is the first potential inhibitor of uric acid crystallization to be described. About 20% of ingested TB is excreted in the urine [17, 18]. TB is currently accepted for use as a diuretic and for its vasodilatory effects, and is therefore used to treat patients with high blood pressure [19]. Previous research indicated that saponins (such as ginseng extract), glycosaminoglycans, and glycoproteins also hindered the crystallization of uric acid [20]. However, the effects of these substances were due to their alteration of the surface tension of water; they are not typical crystallization inhibitors and they do not affect nucleation or crystal growth, in which a substance is adsorbed onto the faces of the crystal. Importantly, these substances also do not elicit dose–response relationships.

Normally, a substance that inhibits the formation of ionic crystals also inhibits crystal dissolution. For example, phytate inhibits the crystallization of calcium salts (oxalate and calcium phosphates), but also inhibits their dissolution [21]. However, we showed here that TB, which was previously determined to inhibit crystallization, promoted crystal dissolution (Fig. 2). In fact, a recent study found that TB and uric acid molecules interacted in solution, to form new tetrameric cluster species [22]. Obviously, the formation of these species would decrease the supersaturation of uric acid and facilitate the dissolution of crystals. It is interesting to note that the action of TB on uric acid stones was completely different from that of NAC. Thus, NAC degraded deposits of organic matter that covered the stone crystals (Fig. 3 C,D), but TB accelerated the dissolution of the crystals (Fig. 3 E,F). Although each substance altered stone shape, TB was more effective at stone dissolution and there were no apparent additive or synergistic effects between these two substances.

We also found that escin had no significant effect on the dissolution of uric acid stones.

It is important to note that there was variability of the dissolution results obtained from the same treatment (Fig. 2), even though we used a stereoscopic microscope to select stone fragments that were as similar as possible. Our SEM results indicated this was likely because stones that appeared macroscopically similar, had major differences in microstructure. In particular, the stones differed in the presence of porosities, the distribution of organic matter and crystal size. In fact, we observed these differences even within an individual calculus. It is also likely that the position of the fragment within the capsule and the flow of liquid around the fragment influenced dissolution.

Our long term (168 h) experiments indicated the presence in controls of small areas of the stones in which there was formation of deposits of sodium/potassium urate crystals (Fig. 3 G). Obviously, the formation of these deposits is a consequence of the high pH and the high uric acid concentration. Unfortunately, these deposits cannot dissolve at high pH. In the presence of TB, as the supersaturation of the urate salt decreases due to the formation of urate-TB clusters, there is reduced formation of these precipitates. In long-term dissolution processes, the formation of urate precipitates can become significant when the pH is very high. In this case, TB can prevent the formation of new stones and can also reduce the formation of insoluble urates, by decreasing the supersaturation of existing urates. In any case, a pH above 7 should be avoided, because this can lead to the formation of hard shells of sodium/potassium urate or apatite salts that coat the stone, making dissolution impossible, and also because uric acid solubility does not significantly increase above that pH value [23]. The formation of insoluble urates (sodium/potassium) [6] will only take place when, due to the concentrations of uric acid, sodium and/or potassium in urine, they exceed the conditions of supersaturation of these salts in this medium. Under conditions of low concentration of uric acid, sodium and potassium in urine (very diluted urine due to high water intake, and/or administration of allopurinol), this supersaturation will not be reached, so this precipitation will not occur. The higher the urinary pH, by increasing the ionization of uric acid [11], the urate crystallization process will also be favored. Theobromine binds to uric acid forming tetrameric species, increasing its solubility [22], so to some extent, it can also prevent the formation of insoluble urates. Fortunately, it seems that the formation of these insoluble urates, which have been observed in "in vitro" experiments [6], and which we have also detected in the study presented in this paper, is not very frequent, although we have detected their presence in kidney stones in some patients (Fig. 3 H).

Finally, our experiments also indicated that use of a higher concentration of TB (80 mg/L) provided no additional benefit.

## Conclusions

When using oral chemolysis to treat uric acid renal stone formers, the addition of an appropriate amount of TB to a basifying therapy, consisting of citrate and/or bicarbonate may improve outcome. TB appears to have two important effects: it prevents the formation of new stones and it increases the dissolution of existing stones. TB can also minimize the formation of insoluble sodium/potassium urate deposits. The U.S. Food and Drug Administration considers TB to be ‘generally regarded as safe’. We therefore suggest that the results presented here should be confirmed by clinical trials.
